# Examining the Results of Virtual Reality-Based Egocentric Distance Estimation Tests Based on Immersion Level

**DOI:** 10.3390/s23063138

**Published:** 2023-03-15

**Authors:** Tibor Guzsvinecz, Erika Perge, Judit Szűcs

**Affiliations:** 1 Department of Information Technology and Its Applications, Faculty of Information Technology, University of Pannonia, Gasparich M. utca 18/A, 8900 Zalaegerszeg, Hungary; szucs.judit@zek.uni-pannon.hu; 2 Department of Basic Technical Studies, Faculty of Engineering, University of Debrecen, Ótemető utca 2, 4028 Debrecen, Hungary; perge@eng.unideb.hu

**Keywords:** desktop display, egocentric distance estimation, depth perception, Gear VR, head-mounted display, human–computer interaction, immersion, virtual reality

## Abstract

Depth perception as well as egocentric distance estimation can be trained in virtual spaces, although incorrect estimates can occur in these environments. To understand this phenomenon, a virtual environment with 11 changeable factors was created. Egocentric distance estimation skills of 239 participants were assessed with it in the range [25 cm, 160 cm]. One hundred fifty-seven people used a desktop display and seventy-two the Gear VR. According to the results, these investigated factors can have various effects combined with the two display devices on distance estimation and its time. Overall, desktop display users are more likely to accurately estimate or overestimate distances, and significant overestimations occur at 130 and 160 cm. With the Gear VR, distances in the range [40 cm, 130 cm] are significantly underestimated, while at 25 cm, they are significantly overestimated. Estimation times are significantly decreased with the Gear VR. When developing future virtual environments that require depth perception skills, developers should take these results into account.

## 1. Introduction

Depth perception is a crucial skill, and egocentric distance estimation is a part of it. This type of distance estimation is defined as the distance from an observer to an object and it is required for several tasks, such as reaching, grasping, or interception [1,2]. Thus, workers in healthcare and military personnel require well-developed depth perception skills [3,4]. It is possible to train these skills both in real environments and in virtual reality (VR) [5]. For example, firefighter and pilot training systems are already developed in virtual environments (VEs) [6,7].

However, interaction and spatial visualization in VEs are not the same as in reality. Human–computer interfaces (such as brain–computer interface (BCI) [8,9,10], eye-tracking [11,12,13], and other similar alternative communication interfaces between human and computer or mobile devices) and interaction can differ between applications [14]. Consequently, among others, I/O devices, the VR engine itself, and humans are integral parts of VR systems, and are equally important [15,16]. Their interaction is of crucial importance [17,18]. Thus, developers must take several factors into account when designing VEs [19], and the users should be the main focus during the process [20]. This is quite important as VR can stimulate cognitive functions, and in turn, visuospatial skills can be enhanced [21,22]. For example, it is possible to affect skills by having various human characteristics, or simply by changing components of VEs, and display devices [23,24,25]. Overall, cognition is a crucial part of VR-based systems [26,27].

As can be suspected, the composition of VEs can also affect distance estimation [28,29], although usually underestimations occur in them [30,31]. Multiple studies mention the importance of binocular disparity regarding distance estimation [32,33,34]. According to Vaziri et al., realism plays an important role in the estimation process [35]. This fact is strengthened by Lappin et al. as they concluded that it can be affected by the scenery itself [36]. Bodenheimer et al. conclude that outdoor environments provide better matching task performance than indoor ones [37]. High graphic quality and immersive depth cues are necessary for distance estimation [38,39]. Similar to the components of VEs, visual cues interact with each other [40]. Perspective cues, such as linear perspective, texture gradient, and foreshortening improved distance perception [41]. Besides graphic quality, other factors were investigated in the literature as well. It was also examined whether the existence of virtual avatars can affect distance estimation [42,43,44]. Luminance contrast can influence it as well [45]. Ground information also plays a critical role [46]: horizontal, vertical lines, or even brick textures can affect distance estimation [47,48]. Contrarily, according to Witmer and Kline, ground texture in VEs does not influence distance estimation [49].

Familiarity can also have an effect on egocentric distance estimation. Interrante et al. created an exact, virtual replica of a real environment, and they concluded that participants did not underestimate distances in it [50]. Similarly, they faithfully modeled replicas of everyday objects, but found no improvement in distance estimation accuracy [51]. Steinicke also tested familiarity with the environment, and received similar results [52]. Thus, if the participants are informed that the VE is an exact replica with the same scale as in reality, estimates are more accurate.

Naturally, egocentric distance itself can have effects on estimation. Several studies show that overestimations occur in peripersonal space (up to about 1 m) [1,53,54,55], although a few conclude otherwise. According to Murgia and Sharkey [28], distances can be underestimated in this space. Contrarily, it is also possible to accurately estimate distances up to 50–55 cm [29,56]. However, in extrapersonal space (about 1–30 m), underestimations can occur [28,53]. Thus, besides the factors of VEs, distances themselves had to be assessed as well.

Based on these facts, we focus on technical and compositional factors regarding a VR system in this research. Therefore, the goal of this study is to assess how immersion level and certain graphical factors affect egocentric distance estimation skills and estimation times of participants at various distances. To do this, we created a VE that can be used with a desktop display and a head-mounted display. Additionally, it is possible to change its composition. For the investigation, we formed the following research question (RQ):

- How are egocentric distance estimation and its times affected by various display parameters and immersion levels?

This article is structured as follows. The materials and methods are shown in Section 2. The VE, data collection, and analysis are detailed in this section. Next, the results are presented in Section 3. All factors have their subsection. Discussion of the results can be observed in Section 4, while Section 5 concludes this study.

## 2. Materials and Methods

To assess the egocentric distance estimation skills in VR, a VE with changeable parameters was developed in Unity (version 2018.4.36f1). This VE has two versions: a PC version and a VR version that runs on Android. The former can be experienced with a desktop display, keyboard, and mouse. In our research, an LG 20M37A (19.5″) desktop display was used. The latter version can be used with the Gear VR head-mounted display and its touchpad on its side. A Samsung Galaxy S6 Edge+ (Samsung Electronics, Suwon, Republic of Korea) was placed into the Gear VR. It is important to note that the participants could not move in the VE. It is only possible to rotate the virtual camera with either the mouse on PC or with head rotations in the case of the VR version. Therefore, the two differences between the two versions are the controls and the level of immersion.

Egocentric distance estimation skills of 239 participants were examined with the aforementioned VE. Overall, their mean age ($M_{age}$) and their standard deviation of age ($SD_{age}$) were the following: $M_{age}=20.65,\;SD_{age}=4.29$. The PC version was used by 157 people at the University of Debrecen ($M_{age}=19.80,\;SD_{age}=2.09$) and the VR one by 72 at the University of Pannonia ($M_{age}=22.51,SD_{age}=6.63$). Regarding participants, 177 were male and 52 were female. Out of males, 128 used the desktop display, while 49 used the Gear VR. For females, 29 and 23 used these two display devices, respectively. The participants came of their own volition and gave verbal consent before the examination commenced. A number (in ascending order) was given to each measurement in the dataset, thus no name was gathered during the process. Based on the data alone, the participants are nonidentifiable.

**Figure 1:**
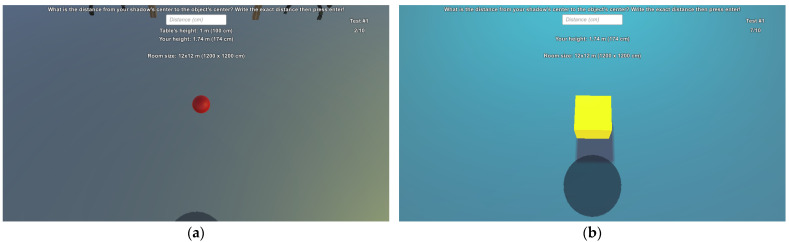

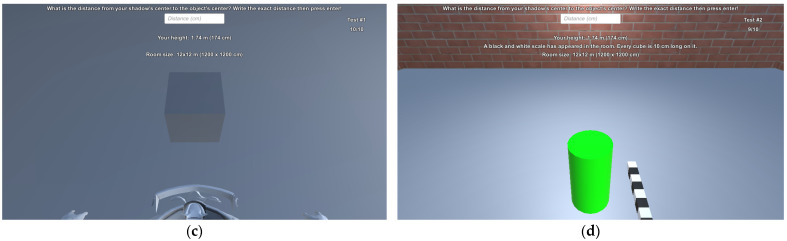
Different randomizations in the VE (not all factors are mentioned in the following description): (**a**) red sphere with a height of 20 cm, furniture, no body, yellow light at right, darker floor, no object shadows, no scale on the ground; (**b**) yellow cube with a height of 30 cm, no furniture, no body, cyan light in front, lighter floor, object shadows, no scale on the ground; (**c**) transparent cube with a height of 40 cm, no furniture, body, white light at right, darker floor, no object shadows, no scale on the ground; (**d**) green cylinder with a height of 50 cm, no furniture, white light in front, lighter floor, no object shadows, scale on the ground.

We assessed the egocentric distance estimation skills of these participants at ten various distances in the range [25 cm, 160 cm] at 15 cm intervals. The order of these distances was randomized, and each one had to be estimated twice. Firstly, all distances had to be estimated without a scale on the ground. After every distance was estimated, then the scale appeared for the same 10 distances, but also in a randomized order. The scale contained 17 cubes 10 cm×10 cm×10 cm, starting from the observer. Information about the measurements was given to each participant before the procedure started: they were briefed about the process and how to enter their estimates, the dimensions of the room, and the scale. They were not informed about the investigated distances and the 15 cm step between them. They were only told that the distance would never be zero. After acknowledging the information, they could start the application. Then, participants had to input the following data either with a keyboard or the touchpad on the Gear VR: their age, gender, height, handedness, average gaming hours per week, whether they have glasses, and whether they have VR experience. After all information was entered, the start button became clickable. By clicking on it, the participant was placed inside the VE, right at its center. The most important datum was the height of the participant, as the virtual camera was placed at the same height in the VE. The room was 12 m wide on both axes. Thus, the distance to the walls was 6 m in every direction. It did not change during the measurements. Examples of this VE with various compositions are shown in Figure 1.

As can be observed, the composition of the VE can be changed. Most of these factors were chosen from the literature mentioned in Section 1, although a few new ones were included in the research as well. Overall, 11 changeable factors were implemented in the VE. Table 1 shows these factors and their possible values.

These factors were randomized in each round, and the whole measurement consisted of 20 rounds. The goal in each round was to estimate egocentric distances to objects that were placed on the VE’s ground in front of the participants. When a round started, the VE began to count estimation times in milliseconds, starting from zero. Regarding estimates, they had to be entered into an input box in the PC version. Contrarily, in the VR version, distance estimation occurred verbally. A researcher immediately typed the received verbal estimates into the dataset. After either the researcher or the participants typed the estimates, participants had to look up to the ceiling with the virtual camera and press enter or the touchpad in order to advance to the next round. In turn, the timer stopped, and the factors were randomized. After each round, a new line of data is written into a *.csv* file. This line contains the previously mentioned information about the participants, the randomized factors, the display device, and the estimation time. Thus, all information about each round is saved in the mentioned file. When a participant completed all 20 rounds, the test that assesses egocentric distance estimation ended.

After collecting the data, the two (PC and VR) datasets were merged and checked for errors. Then, their distributions were analyzed with the Shapiro–Wilk normality test using the statistical program package R. The test statistic (W) and the probability of type I error (p) are returned by this test. Depending on the results of this test, either parametric or nonparametric tests should be used when comparing the results to each other. After using the Shapiro–Wilk normality test, the results showed that the distributions of the following groups were non-Gaussian: $Distance_{PC}(W=0.88,p<0.001)$, $Time_{PC}(W=0.71,p<0.001)$, $Distance_{VR}(W=0.79,\;p<0.001)$, $Time_{VR}\left(W=0.68,\;p<0.001\right)$. Therefore, nonparametric tests should be used in each case for further analyses. We used the Wilcoxon signed rank test when comparing estimates to actual distances, while we used its rank sum variant when either estimates or their times were compared between platforms. Both return the probability of type I error (p) and their respective test statistics (V in the case of the former and W in the case of the latter). Regarding these tests, V also means the sum of the ranks of the pairwise differences, while W also means the smaller of rank totals. For the analyses, an α=0.05 was selected. When comparing estimates to actual distances, estimates within an error margin of ±10% were considered accurate.

## 3. Results

**Figure 2:**
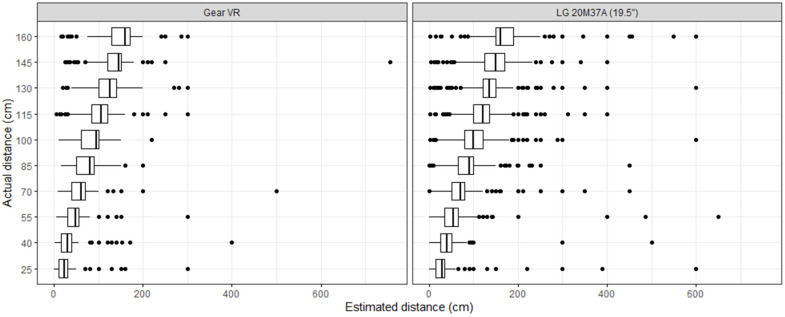
Distance estimates grouped by display devices.

**Figure 3:**
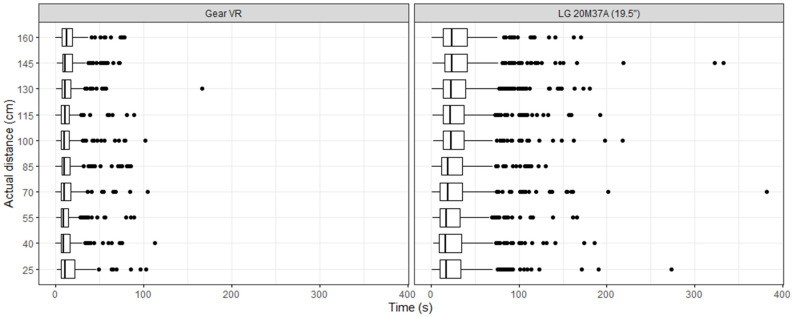
Estimation times grouped by display devices.

The results of the investigation are presented in this section. First, the whole dataset was examined and the estimates were investigated. After the analysis concluded, the estimation times were assessed. The results are shown with boxplots: dots are outlier values; the left and right sides of the boxes show the lower and upper quartiles, respectively; and the vertical line in the middle is the median. Naturally, the minimum and maximum values can also be read from the boxplots. The entire dataset can be seen in Figure 2 and Figure 3.

When the actual distances were compared to estimates in the PC version, each of them was overestimated, although not all were significant. In the range [40 cm, 160 cm], the overestimates were smaller than 10%. Those that were significant could be observed at 130 cm (V=20,885, p=0.017) and 160 cm (V=21,559, p=0.014). In the VR version, underestimates were observed at all distances except for 25 cm. Although significant, it was an overestimation. The results for VR follow: 25 cm (V=2610, p=0.001), 40 cm (V=1426, p<0.001), 55 cm (V=1718, p<0.001), 70 cm (V=1301, p<0.001), 85 cm (V=2224, p<0.001), 100 cm (V=976, p<0.001), 115 cm (V=2893, p<0.001), and 130 cm (V=2334, p<0.001). No significant differences were found at 145 and 160 cm. Afterward, the same distances were compared on both platforms. The two smallest (but still significant) differences were at 145 cm (W=25,514, p=0.026) and 160 cm (W=25,814, p=0.014). The differences are strongly significant for the remaining distances (27,052≤W≤28,921, p<0.001).

Regarding estimation times, all are significantly different between the platforms (29,260≤W≤35,550, p<0.001). By using the Gear VR, estimation times were significantly decreased by 35.73–57.14% depending on the distances. The largest decrease was at 115 cm, while the smallest one was at 25 cm.

To fully understand the factors, each of them was evaluated. The following 11 subsections describe the results grouped by the aforementioned examined factors. A summary can be read at the beginning of each subsection, while they are elaborated on later. Each elaboration is structured as follows. First, the estimates were compared to the actual distances on PC, then on VR. Afterward, the estimates were compared between the two versions. Lastly, estimation times were also compared between the versions.

### 3.1. Object Type

The results are grouped by the three object types and display devices. As was mentioned, the possible object types were cube, cylinder, and sphere. Regarding object types, accurate or overestimates can be observed in the case of the PC version. In the VR version, underestimates can be found. There were several significant differences found between the versions, although it should be noted that the estimation process was faster on VR than on PC.

#### 3.1.1. Cube

When comparing the actual distances to the estimated ones using the desktop display, no significant differences could be found among them. Still, they were either accurate or overestimated. It is different for the case of the Gear VR version. Significant differences were found in the range [40 cm, 115 cm] (34≤V≤291, p<0.001). All distances were underestimated. When the estimated distances were compared between the two display devices, significances occurred in the range [40 cm, 130 cm] (2672.5≤W≤3889, p≤0.03). Regarding egocentric distance estimation times, significant differences were found at all distances (2662≤W≤4450, p<0.017). Estimation was quicker on VR by 49.95%, on average.

#### 3.1.2. Cylinder

If the actual distances were compared to the estimated ones in the PC version, two significant differences could be found in the case of cylinders. These differences were at 25 cm (V=4235,p<0.001) and at 75 cm (V=1096.5, p=0.005). The former was overestimated by 39.37%, while the latter was underestimated by 7.69%. In the case of the VR version, significant differences were found in the range [25 cm, 130 cm] (58.5≤V≤354.5, p≤0.048). Here, all were underestimates. When the distances were compared between the platforms, significant differences could be observed in the range [25 cm, 55 cm] and [100 cm, 130 cm] (3117≤W≤4208.5, p<0.013). Similarly, estimation was significantly quicker on VR (2694≤W≤4873, p<0.019) by 46.99%, on average.

#### 3.1.3. Sphere

In the case of spheres, there were two significant differences between actual distances and estimated ones in the PC version. These differences could be observed at distances of 115 cm (V=3865.5, p=0.040) and 160 cm (V=3918.5, p=0.015). An increase of 6.39% was found in the case of the former, while an increase of 9.76% was found in the case of the latter. There were two significant differences as well in the VR version. They could be observed at 70 cm (V=70.5, p<0.001) and 85 cm (V=310.5, p=0.002). These were underestimates of 17.50%, and 14.85%, respectively. Still, even the nonsignificant differences were underestimated. When comparing the distance estimates between the two versions, significant differences could also be observed at 70 cm (W=2743, p=0.001) and 85 cm (W=3774.5, p=0.003). Estimation was also significantly faster on VR at all investigated distances (3527≤W≤5082, p<0.001) by 48.81%, on average.

### 3.2. Object Height

Next, the height of objects was investigated. As was mentioned, the heights of 20, 30, 40, and 50 cm were examined. On PC, except for object height of 20 cm, overestimates could be observed. Contrarily, in the VR version, underestimates could be found in the case of all heights. Similarly to object type, the use of the Gear VR significantly decreases estimation time.

#### 3.2.1. Object Height 20 cm 

Regarding the PC version, significant differences could be observed at distances of 40 cm (V=866.5, p=0.011), 70 cm (V=506.5, p=0.017), 130 cm (V=1758.5, p=0.012), and 160 cm (V=2211, p<0.001). It is interesting to note that below 70 cm, underestimates occurred, while overestimates could be observed above this distance. Regarding the VR version, significant differences arose in the range [55 cm, 100 cm] (76.5≤V≤115.5, p<0.01). Except for 25 cm, each distance was either accurate or underestimated. When the distance estimates were compared between the two platforms, significant differences arose at 55 cm (W=1733.5, p=0.010), 85 cm (W=2004, p=0.016), 100 cm (W=2263.5, p=0.003), 130 cm (W=1895, p=0.011), 145 cm (W=1904.5, p=0.048), and at 160 cm (W=2442, p=0.038). Estimation was significantly faster using the Gear VR (1979≤W≤2908, p<0.001) by 53.44%, on average.

#### 3.2.2. Object Height 30 cm 

On PC, there was one significant observation between actual distances and estimates. This was at the egocentric distance of 130 cm (V=1825, p=0.008). Here, overestimates occurred at each distance. On VR, significant differences were in the range [25 cm, 70 cm] and [100 cm, 130 cm] (74.5≤V≤248, p<0.031). Except for 145 and 160 cm, every distance was underestimated. When comparing the distance estimates between the two platforms, the following five significant differences could be observed: 25 cm (W=1851.5, p<0.001), 55 cm (W=2303.5, p<0.033), 70 cm (W=1909,p=0.004), 115 cm (W=1700.5,p=0.024), and 130 cm (W=2735.5, p<0.001). As noted previously, estimation was significantly faster on the Gear VR (1712≤W≤3300, p<0.027) by 47.02%.

#### 3.2.3. Object Height 40 cm 

Contrary to the previous investigations, there are no significant differences in the PC version when actual distances are compared to estimates. Still, distances were overestimated. In the VR version, significant differences can be found in the range [40 cm, 115 cm] (41≤V≤143.5, p<0.014). The distances were underestimated in this version. When the estimates were compared among the two versions, five significant differences can be observed. These ones are at 40 cm (W=1835.5, p<0.001) and in the range [70 cm, 115 cm] (1123≤W≤1643, p<0.037). Similar to the previous object height, estimation was also significantly faster with the Gear VR at all distances (1187≤W≤2500, p<0.019) by 43.96%, on average.

#### 3.2.4. Object Height 50 cm 

Similar to the previous examination, no significant differences could be found in the PC version if the distances were compared to the estimates. Distances were overestimated at most distances; the exceptions were 85 and 130 cm. In the case of the VR version, these significant differences were in the range [55 cm, 115 cm] (67≤V≤114.5, p<0.015) and at 145 cm (V=183.5, p=0.031). As noted previously, distances were underestimated in this version. When the estimates between the two versions were compared, four significant differences could be found. These are at distances of 40 cm (W=1386, p=0.036), 55 cm (W=1859, p=0.008), 85 cm (W=1714, p=0.034), and at 115 cm (W=2240, p<0.001). With the Gear VR, estimation was also significantly faster in the range [40 cm, 160 cm] (1557≤W≤2634, p<0.001) by 47.55%, on average. As can be seen, no significant difference was found between times at 25 cm.

### 3.3. Object Color

Next, the color of objects was investigated: black, blue, green, red, transparent, white, and yellow. Regarding these colors, accurate or overestimates could be observed on PC. In the Gear VR version, underestimates could be found. However, the results show that significant differences in estimation times can be affected by object color.

#### 3.3.1. Black

In the case of black objects on PC, three significant differences were found when the actual distances were compared to estimates. These were at 70 cm (V=285, p=0.014), 115 cm (V=512, p=0.039), and 160 cm (V=786, p=0.018). Overall, either accurate or overestimates were found. In the VR version, significant differences could be found at distances of 55 cm (V=56, p=0.012), 85 cm (V=42, p=0.006), 100 cm (V=0, p=0.002), and 130 cm (V=9.5, p=0.022). All were underestimates. Only the 25 cm was overestimated. When the estimates were compared between the two versions, significant differences were observed at 100 cm (W=636, p=0.002), 130 cm (W=583, p=0.006), and 160 cm (W=821, p=0.009). Estimation was only faster in the range [55 cm, 160 cm] when the Gear VR was used (470≤W≤1032, p<0.019). The process was faster by 46.20%, on average.

#### 3.3.2. Blue

Regarding blue objects, the number of significant differences was smaller. There were no such observations in the PC version. Still, either accurate or overestimates occurred. On VR, they were found at 70 cm (V=8, p=0.003) and 100 cm (V=28.5, p=0.043). Similar to the previous color, all were underestimated except for 25 cm. When the results were compared between the versions, two significant differences were found as well, one at 40 cm (W=706, p=0.031) and another at 70 cm (W=915, p=0.001). Distance estimation was significantly faster at 25 cm (W=766, p=0.004) and in the range [55 cm, 160 cm] (584≤W≤963, p<0.028) using the Gear VR by 43.94%, on average.

#### 3.3.3. Green

Similar to the previous examination, there were no significant differences in the PC version in the case of green objects. Even though not significant, either accurate or overestimates occurred. In the VR version, four could be observed: at 40 cm (V=14, p=0.016), 70 cm (V=11, p=0.005), 85 cm (V=50, p=0.001), and 100 cm (V=21, p=0.028). Every distance was underestimated, even the nonsignificant ones. Four significant differences could be observed between the two versions: at 40 cm (W=872.5, p=0.002), 55 cm (W=368, p=0.042), 85 cm (W=1045.5, p=0.012), and 100 cm (W=600.5, p=0.014). The distances of 25, 70, and 145 cm did not yield significant differences in time between the platforms. In the case of the remaining distances, estimation was significantly faster with the Gear VR (350≤W≤1142,p<0.039), by 44.55%, on average.

#### 3.3.4. Red

There was only one significant difference in the PC version regarding red objects, which could be observed at 40 cm (V=126.5, p=0.009). Still, every estimate was accurate or an overestimate. The following significant differences were found in the VR version at [40 cm, 100 cm] (5≤V≤42, p<0.020) and at 145 cm (V=32, p=0.011). Each distance was underestimated. Between the two versions, three significant differences were found: at 70 cm (W=630.5, p<0.001), 130 cm (W=774.5, p=0.020), and 145 cm (W=671.5, p=0.004). Estimation is also faster by 55.36%, on average, with the Gear VR at every investigated distance except for 55 cm (619≤W≤997, p<0.006).

#### 3.3.5. Transparent

In the case of transparent objects, only one significant difference was found in the PC version: at 25 cm (V=651, p=0.031), which was an overestimate. The remaining ones were also overestimates or accurate. In the VR version, the following three significant differences were found: at 55 cm (V=31, p=0.032), 70 cm (V=27, p=0.006), and 100 cm (V=10.5, p=0.009). It should be noted that 25, 40, 145, and 160 cm were overestimated, the remaining ones were underestimated. Five significant differences were found between the versions: at 25 cm (W=662.5, p=0.024), 55 cm (W=765, p=0.033), 70 cm (W=550, p=0.008), 100 cm (W=655.5, p=0.031), and 115 cm (W=719.5, p=0.013). Estimation is only made faster in the range [55 cm, 160 cm] (409≤W≤841, p<0.024) by using the Gear VR, meaning that estimation time decreased by 46.34%, on average.

#### 3.3.6. White

Regarding white objects, overall six significant differences were found. One such difference could be seen at 100 cm (V=315, p=0.035) in the PC version. Three of them were observed at 25 cm (V=13, p=0.044), 55 cm (V=19.5, p=0.001), and 145 cm (V=36, p=0.017) in the VR version. All distances were underestimated except for 40 and 160 cm in this version. Lastly, two significant differences were found at 25 cm (W=471, p=0.028) and 55 cm (W=587, p=0.042) when the two versions were compared. The use of the Gear VR significantly decreases estimation time by 51.13%, on average, at all distances (370≤W≤918, p<0.033).

#### 3.3.7. Yellow

When comparing estimates to actual distances in the case of yellow objects, no significant differences could be found in the PC version. Estimates were either accurate or overestimated. In the VR version, significant differences were observed in the range [40 cm, 70 cm] (22≤V≤34, p<0.040). All distances were underestimated on VR. Between the two versions, these differences were at 25 cm (W=461, p=0.021), 40 cm (W=618.5, p=0.041), and 115 cm (W=653, p=0.045). Except for distances of 25, 55, and 85 cm, estimation times were significantly decreased by 46.91% with the use of the Gear VR (599≤W≤849, p<0.004).

### 3.4. Shadows in the Scene

In most cases, the distances were also accurate or overestimated on PC. Contrarily, they were underestimated in the VR version. Similar to the previous investigations, estimation times can be significantly decreased by using the Gear VR.

#### 3.4.1. No Shadows

On PC, there was only one significant difference at 130 cm (V=6115, p<0.001). All were overestimated except for 70 cm. On VR, significant differences were observed in the range [40 cm, 130 cm] (275≤V≤610, p<0.021); 25 cm was overestimated, while the remaining ones were underestimated. Between the two versions, there were significant differences in the range [25 cm, 130 cm] (5567≤W≤7807.5, p<0.040). By using the Gear VR, estimation times were significantly decreased by 49.84%, on average, at all distances (6757≤W≤10,516,p<0.001).

#### 3.4.2. Shadows

Similar to the previous examination, only one significant difference could be found in the PC version. However, it was at 160 cm (V=4940, p=0.034). All distances were accurate or overestimated. In the case of the VR version, significant differences could be observed in the range [25 cm, 130 cm] (193≤V≤936.5, p<0.016) and at 160 cm (V=434, p=0.012). All distances were underestimated on VR. When the estimates were compared between the two versions, significant differences could be found in the range [25 cm, 115 cm] (6933≤W≤8660.5, p<0.025) and at 160 (W=6427.5, p=0.002). Except for 25 cm, the use of the Gear VR significantly decreased estimation times by 47.34%, on average, at the other distances (6165≤W≤10,488, p<0.001).

### 3.5. Light Location Relative to the Observer

Next, the light location relative to the observer was assessed. Locations could be back, front, left, and right. Similar to the previous factor, accurate or overestimates occurred with a desktop display. With the Gear VR, distances were usually underestimated. However, when the light was in front of the observer, overestimates could be found in the dataset. Still, by using the Gear VR, estimation was significantly faster at almost all distances.

#### 3.5.1. Back

When the light was behind the observer, two significant differences could be observed between actual distances and estimates in the PC version: at 40 cm (V=769.5, p=0.031) and 85 cm (V=1210.5, p=0.048). All were accurate or overestimated. On VR, five significant differences could be seen: at 55 cm (V=74.5, p=0.001), 70 cm (V=34.5, p<0.001), 85 cm (V=137, p=0.049), 115 cm (V=206, p=0.027), and 130 cm (V=136, p=0.047). Overall, only 25 cm was overestimated, and the others were underestimated. There are four significant differences between the versions: at 25 cm (W=1602, p=0.043), 55 cm (W=2209.5, p=0.003), 70 cm (W=1574.5, p=0.016), and 115 cm (W=1841.5, p=0.033). Except for 25 cm, estimation times were significantly faster in the case of the Gear VR by an average of 50.08% (1685≤W≤2418, p<0.039).

#### 3.5.2. Front

If the light is in front of the observer, only one significant difference could be observed in the PC version: 130 cm (V=1624, p=0.002). Similarly, all estimates were either accurate or overestimated. On VR, significant differences could be found in the range [70 cm, 100 cm] (27.5≤V≤237.5, p<0.009). Overestimates can be found at 25 and 40 cm, while at the others, distances were accurately or underestimated. Between the two versions, significant differences could be found at 85 cm (W=2227, p=0.019), 100 cm (W=1794.5, p=0.004), and 130 cm (W=1904.5, p=0.002). As noted previously, estimating distances was significantly faster on the Gear VR (1622≤W≤2830, p<0.001) by an average of 52.40%.

#### 3.5.3. Left

Regarding the light that is left of the observer, no significant differences were found in the PC version. All estimates were either accurate or overestimated. On VR, significant differences could be found in the range [25 cm, 100 cm] (29≤V≤110, p<0.011) and at 130 cm (V=76, p=0.035). All distances were underestimated on VR. When the two versions were compared, differences arose in the range [25 cm, 70 cm] (1482.5≤W≤1740, p<0.006) and at 100 cm (W=2188.5, p=0.024). Estimation was significantly faster with the Gear VR (1356≤W≤2715, p<0.010) by 41.83%, on average.

#### 3.5.4. Right

If the light was right of the observer, significant differences were found at 55 cm (V=941.5, p=0.035), 115 cm (V=2289, p=0.042), and 130 cm (V=1328.5, p=0.028) in the PC version. In the case of the VR version, these differences could be observed in the range [40 cm, 115 cm] (75≤V≤168.5, p<0.027). Every distance was underestimated using the Gear VR. When the estimates between the versions were compared, significant differences were found at 40 cm (W=2310, p=0.006), 70 cm (W=2024, p=0.016), 85 cm (W=1912, p=0.004), 100 cm (W=1711.5, p=0.018), 115 cm (W=2359, p<0.001), 130 cm (W=2059, p=0.013), and 160 cm (W=1555, p=0.048). Except for 25 cm, estimating distances was significantly faster with the Gear VR (1847≤W≤2666, p<0.002) by 47.23%, on average.

### 3.6. Light Color

The color of light was investigated next. The colors could be cyan, green, white, or yellow. According to the results, light color can usually affect estimates at 25 cm on VR. Otherwise, distances are underestimated. On PC, they are still either accurate or overestimated. Estimation times were also significantly decreased by the Gear VR; 25 cm was an exception in the case of two colors.

#### 3.6.1. Cyan

In the case of cyan light, no significant differences were found on PC, although each distance was either accurate or overestimated in this version. In the VR one, significant differences were observed in the range [55 cm, 85 cm] (51.5≤V≤88, p<0.004) and at 160 cm (V=124.5, p=0.026). All distances were underestimated on VR. When the estimates between the two versions were compared, significant differences could be observed at 25 cm (W=1805.5, p=0.032), 55 cm (W=1860, p=0.024), and 70 cm (W=1790, p=0.024). Similar to the previous factors, using the Gear VR made estimation times significantly faster (1634≤W≤3041, p<0.008) at all distances except for 25 cm by an average of 47.52%.

#### 3.6.2. Green

Similar to the cyan light color, no significant differences were found in the PC version between actual distances and estimates. Still, every distance was either accurate or overestimated. In the case of the VR version, they could be observed in the range [40 cm, 130 cm] (34≤V≤241.5, p<0.037). Estimates were accurate at 25 cm, while the others were underestimated. When compared between the two versions, these differences arose in the range [25 cm, 55 cm] and [85 cm, 130 cm] (1368.5≤W≤2201, p<0.048). Similarly, there was no significant difference in estimation time at 25 cm between the platforms. Estimation was made faster with the Gear VR at the others (1773≤W≤2599, p<0.001) by 47.99%, on average.

#### 3.6.3. White

On PC, even though accurate or overestimates occurred, no significant differences were found between actual and estimated distances in the case of white light. On VR, significant differences occurred at 40 cm (V=162, p=0.007), 55 cm (V=193, p=0.045), 70 cm (V=47, p<0.001), and 100 cm (V=30, p<0.001). While all distances were underestimated, 25 cm was overestimated. Several significant differences can be observed between platforms. These were at 40 cm (W=1998.5, p=0.008), 55 cm (W=1901, p=0.018), 70 cm (W=1883.5, p=0.001), 100 cm (W=2381.5, p<0.001), and 130 cm (W=1576, p=0.030). As opposed to the previous colors, estimation at all distances was made faster by the Gear VR (1813≤W≤2458, p<0.010). This decrease in time was 46.74%, on average.

#### 3.6.4. Yellow

Regarding yellow light, significant differences could be found in the PC version at 115 cm (V=1722.5, p=0.004) and 145 cm (V=2220.5, p=0.029). As noted previously, estimates were either accurate or overestimates. On VR, these significant differences could be observed in the range [25 cm, 130 cm] (28≤V≤251, p<0.019). All distances were underestimated in this version. Between the two platforms, significant differences were in the range [25 cm, 55 cm] (1266.5≤W≤1833.5, p<0.035), 85 cm (W=1906, p=0.042), and in the range [115 cm, 145 cm] (1164≤W≤2419.5, p<0.007). Estimation at all distances became faster with the Gear VR (1226≤W≤2886,p<0.020) by 51.90%, on average. As can be seen, this color decreased estimation time the most.

### 3.7. Body

Whether the user had a virtual body or a shadow was investigated next. As with previous factors, distances were either accurate or overestimated with or without a body. Distances were underestimated with the Gear VR and estimation was also significantly faster with it.

#### 3.7.1. Having a Body

On PC, significant differences were found in estimates at 40 cm (V=3589, p=0.037), 100 cm (V=5801, p=0.012), 115 cm (V=7278.5, p=0.011), 130 cm (V=5340.5, p=0.003), and 160 cm (V=7121.5, p=0.006). Distances were mostly overestimated on PC, and the average estimate was 102.19 cm. Contrarily, on VR, significant differences were found in the range [25 cm, 145 cm] (279≤V≤804.5, p<0.043). Distances were underestimated on VR, and the average estimate was 83.67 cm. Between the two platforms, significant differences were found at the same distances as in the VR version (6161≤W≤8695.5, p<0.021). Distance estimation was significantly faster with the Gear VR (6762≤W≤10,510, p<0.001). On average, it was faster by 46.98%.

#### 3.7.2. Having Only a Shadow

Without a body, the estimates on PC were 93.02 cm on average, and a significant difference was found at 70 cm (V=3176.5, p=0.011). On VR, significant differences were observed in the range [40 cm, 130 cm] (190≤V≤677, p<0.034). The average was 85.91 cm. When the two versions were compared, they were found in the range [40 cm, 70 cm] and [100 cm, 130 cm] (6272.5≤W≤7952, p<0.036). The Gear VR made estimation significantly faster at every distance (6391≤W≤9346, p<0.001). The decrease in time was 50.04%.

### 3.8. Furniture

It was investigated next whether extra furniture was present. As noted previously, distances were either accurate or overestimated on PC, and they were underestimated on VR. The use of the Gear VR also significantly decreases estimation times at all distances.

#### 3.8.1. Having Furniture in the Room

When having furniture in the room, two significant differences could be observed between distances in the PC version: at 70 cm (V=4661, p=0.013) and 160 cm (V=6000, p=0.026). All estimates were either accurate or overestimated. In the VR version, significant differences were found in the range [25 cm, 145 cm] (177.5≤V≤770.5, p<0.033). On VR, all estimates were accurate or underestimated. Between the two versions, significant differences were observed in the range [25 cm, 130 cm] (6037.5≤W≤8120, p<0.022) and at 160 cm (W=7081.5, p=0.007). By using the Gear VR, distance estimation time was made significantly faster (6842≤W≤9798, p<0.001) by 48.18%, on average.

#### 3.8.2. Having No Furniture in the Room

Without extra furniture, significant differences were found at 115 cm (V=7663.5, p=0.010) and 130 cm (V=6260.5, p=0.003) on PC. Still, having no furniture did not make a difference, since all estimates were either accurate or overestimated. Contrarily, in the VR version, significant differences were found in the range [25 cm, 130 cm] (239.5≤V≤815, p<0.018). As with furniture, all estimates were accurate or underestimated. When the estimates were compared between the platforms, the same distances yielded significant differences as previously observed (6133≤W≤8419.5, p<0.022). Using the Gear VR also made estimation faster (6339≤W≤9990,p<0.001) by 49.67%, on average.

### 3.9. Floor Texture

Floor texture was examined next. This factor had three possible values: brick textures, brighter floor, and darker floor. Brick textures had the largest effect on PC since they yield the most accurate results. However, they had no effect in the VR version as the distances were still underestimated. Brighter and darker floors did not present different results than the factors investigated previously. The Gear VR still significantly decreased estimation times.

#### 3.9.1. Brick Floor

On PC, there was no significant difference between distances and estimates when a brick floor texture was used. Only 25 cm was overestimated, the others were accurate. On VR, significant differences were observed in the range [25 cm, 100 cm] (68≤V≤307.5, p<0.017) and at 130 cm (V=207.5, p=0.048) and 145 cm (V=295, p=0.019). Still, distances were underestimated. When the estimates were compared between the versions, significant differences were observed in the range [25 cm, 115 cm] (2623.5≤W≤3855.5, p<0.048) and at 145 cm (W=2372, p=0.046). As previously noted, estimation times were significantly decreased by the use of the Gear VR (2990≤W≤3853, p<0.011). An average decrease of 51.32% could be observed.

#### 3.9.2. Brighter Floor

When a brighter floor was used, three significant differences could be found on PC. These were observed at 85 cm (V=2624.5, p=0.033), 130 cm (V=2765, p=0.001), and 160 cm (V=3327.5, p=0.010). Distances were overestimated. On VR, significant differences were observed in the range [40 cm, 130 cm] (45.5≤V≤325, p<0.047). Distances were either accurate or underestimated. When the estimates were compared between the versions, significant differences could be observed at 40 cm (W=3071, p<0.001), 55 cm (W=3759, p=0.001), 70 cm (W=3612.5, p=0.001), 115 cm (W=2802, p=0.022), 130 cm (W=3625.5, p<0.001), and 160 cm (W=2777, p=0.031). Estimation times are significantly decreased by using the Gear VR (2867≤W≤4717, p<0.016). The average decrease was 44.12%.

#### 3.9.3. Darker Floor

On PC, significant differences were found at 40 cm (V=1373.5, p=0.045) and 70 cm (V=1477.5, p=0.031). Aside from the significant differences, distances were either correctly or overestimated. Contrarily, in the VR version, distances were underestimated, and significant differences were found at 25 cm (V=264.5, p=0.030) and in the range [55 cm, 115 cm] (74≤V≤443, p<0.033). Between distances among platforms, significant differences were found at 25 m (W=2596.5, p=0.049) and in the range [85 cm, 130 cm] (2706.5≤W≤3970, p<0.014). The Gear VR significantly (2942≤W≤4974, p<0.001) decreased estimation times by 49.42%, on average.

### 3.10. Wall Texture

Next, wall textures were investigated. There were three variations: gray brick texture, no wall texture, and red brick texture. Gray brick textures produced the most accurate results on PC. Otherwise, the distances were overestimated. On VR, distances were still underestimated. The Gear VR also significantly decreased estimation times.

#### 3.10.1. Gray Brick Texture

In the case of gray brick wall textures, one significant difference was found on PC at 130 cm (V=3250.5, p=0.003). However, while 25 cm was overestimated, the remaining nine distances were accurately estimated. In the VR version, significant differences were observed in the range [25 cm, 100 cm] (104≤V≤252.5, p<0.013). All distances were either accurate or underestimated. Between the two platforms, significant differences were found at 25 cm (W=2619.5, p=0.007) and in the range [55 cm, 130 cm] (2471.5≤W≤3794, p<0.048). Estimation times were significantly faster (2882≤W≤4576, p<0.001) with the Gear VR. They became faster by 51.90%.

#### 3.10.2. No Wall Texture

Significant differences were not found in distances on PC when the walls had no texture. Still, observers were accurate at seven distances (55, 85, 100, 115, 130, 145, and 160 cm), while overestimates were made at three (25, 40, and 70 cm). On VR, significant differences were observed in the range [40 cm, 130 cm] (2.5≤V≤462.5, p<0.034). When the estimates were compared between the two platforms, significant differences were found in the range [40 cm, 70 cm] and [100 cm, 115 cm] (2292≤W≤5439.5, p<0.010). By using the Gear VR, distance estimation times were decreased significantly (2022≤W≤6503, p<0.017) by 44.20%, on average.

#### 3.10.3. Red Brick Texture

If red brick textures were used on the walls, significant differences could be observed at 70 cm (V=1939, p=0.010) and 160 cm (V=2756.5, p=0.015) on PC. Similar to having no textures, observers were accurate at seven distances (40, 70, 85, 115, 130, 145, and 160 cm) and overestimated distances at three (25, 55, and 100 cm). Contrarily, on VR, these differences could be found in the range [55 cm, 100 cm] (18≤V≤192, p<0.003) and 130 cm (V=276.5, p=0.017); 25 and 40 cm were overestimated, while the remaining ones were either accurate or underestimated. When the estimates were compared between the platforms, significant differences arose at 25 cm (W=3159.5, p=0.029), 55 cm (W=3279.5, p=0.005), 100 cm (W=2934.5, p<0.001), and 130 cm (W=3440.5, p=0.004). Estimation times became significantly faster (2719≤W≤5011, p<0.010) by 49.15%, on average, with the Gear VR.

### 3.11. Existence of a Scale

Lastly, the existence of a scale was assessed. Whether scales exist in the scene or not, distances were either accurate or overestimated on PC, while they were either accurate or underestimated on VR. The use of the Gear VR still significantly decreased estimation times.

#### 3.11.1. Not Having a Scale

If no scale was present, six significant differences could be observed on PC: in the range [25 cm, 100 cm] (2532≤V≤4271, p<0.025). All distances were accurate or overestimated. On VR, eight significant differences could be found in the range [25 cm, 130 cm] (2532≤V≤4271, p<0.025). The distances were either accurate or underestimated. There were seven significant differences between the platforms: [25 cm, 115 cm] (6622≤W≤7070.5, p<0.019). Using the Gear VR, estimation times significantly decreased (6764≤W≤8751, p<0.016) by 50.07%, on average.

#### 3.11.2. Having a Scale

If there was a scale, significant differences were found at all investigated distances on PC (4561≤V≤8657, p<0.009). Distances were either accurate or overestimated. On VR, one significant difference was found at 70 cm (V=281.5, p=0.002). Distances were accurate or underestimated. When the estimates were compared between the two platforms, significant differences were found at all distances except for 160 cm (6598.5≤W≤8287.5, p<0.039). Estimation times were significantly decreased (7488≤W≤9161, p<0.001) using the Gear VR. The decrease was 46.16%, on average.

## 4. Discussion

Based on the results, it can be said that our RQ was answered. Similar to previous studies reported in the literature [32,33,34], we also highlight the fact that providing binocular disparity is important when estimating distances. Its existence can change an overestimation to an underestimation. Consequently, using the Gear VR resulted in underestimates in almost all cases, except at a distance of 25 cm. Still, it is possible to accurately estimate distances without binocular disparity depending on the composition of the VE. According to Naceri et al. and Viguier et al. [29,56], accurate estimates can occur up to 50–55 cm. They also conclude that errors increase with distance. Our study also strengthens this fact, although if we consider estimates that are in ±10% of the actual distances to be accurate, it becomes possible for them to be also accurate at larger distances, such as 160 cm. Still, those who used the Gear VR became more accurate when scales were present in the VE than those who used a desktop display. However, having scales did not decrease estimation times between platforms as much as when not having scales.

Several studies also mention the importance of ground information [46,47,48]. This is also true in our case. Our results show that desktop display users were more affected by brick ground textures than Gear VR users. By having a brick texture on the ground, significant differences between estimates and actual distances were eliminated when the desktop display was used. Contrarily, these differences remained in the case of the Gear VR, although smaller underestimates occurred. Regarding estimation times, brick textures provided the largest decrease between the two platforms, making the Gear VR users more confident in the estimation process.

As was mentioned, luminance contrast was also investigated and reported in the literature [45]. While we did not investigate it, we examined light location and color. On PC, these have smaller effects, since there are only a few significant differences between actual distances and estimates. Contrarily, the number of significant differences is larger in the VR version. On another note, the effect of a virtual body is divisive. Creem-Regehr et al. conclude that it has no effect [57], but others observed it can affect egocentric distance estimation [42,43,44,58]. According to them, this is due to having a dynamic body that follows the motion of the user. Still, some studies even mention that distance estimates were not improved significantly when a tracked virtual body was present [59,60,61]. In our case, participants either had a static virtual body or a shadow. They overestimated distances when having a virtual body, and were more accurate when they only had a circular shadow. When using the Gear VR, their estimates also became a little more accurate and faster without a body.

In addition to these, several other investigated factors can affect egocentric distance estimation and its time in VEs. Each has a greater effect in the case of one of the two investigated display devices. Thus, one of the most important factors is the immersion level. Not only does it change how distances are perceived, but it also affects estimation times. Therefore, the use of the Gear VR makes interaction significantly faster with its touchpad on the side. These results also strengthen the fact that a VR system is complex, and humans, I/O devices, and the engine itself play an important role in it. Thus, the composition of VEs is crucial regarding depth perception.

## 5. Conclusions

To assess egocentric distance estimation skills of people, a VE was created with 11 changeable parameters or factors. Since display devices also play an important role in depth perception, participants either used a desktop display or the Gear VR to test their egocentric distance estimation skills. Overall, on a desktop display, users are more likely to accurately estimate or overestimate distances to objects. Significant overestimations occur at 130 and 160 cm. Contrarily, by using the Gear VR, egocentric distances in the range [40 cm, 130 cm] are usually underestimated. While underestimates can still occur, out of the investigated distances, objects 25 cm away from the observers are most frequently overestimated with the mentioned head-mounted display. Regarding estimation times, they significantly decreased when the Gear VR was used. The decrease was between 35.73 and 57.14% depending on the distances. The number of underestimates, accurate estimates, and overestimates at each investigated distance are shown in Table 2. In the table, desktop display (DD), Gear VR (GVR), underestimate (UE), accurate estimate (AE), and overestimate (OE) are abbreviated.

Still, it can be seen that the estimates and times change significantly depending on the investigated factors. Even if investigated by the 11 factors, distances are still underestimated with the Gear VR. Estimating distances at 25 cm with the Gear VR produced the most diverse results. With the said head-mounted display, estimation times are also significantly decreased. Some factors make the differences in estimation times nonsignificant in the case of 25 cm. Thus, in conclusion, estimating objects at 25 cm proved to be the most interesting in the dataset. When developing future VEs that require depth perception skills, developers should take these results into account. Additionally, if speed is important in the VE, a head-mounted display should be used.

## Figures and Tables

**Table 1:** Factors and their possible values.

Factor	Possible Values
Object type	Cube; Cylinder; Sphere
Object height	20; 30; 40; 50 cm
Object color	Black; Blue; Green; Red; Transparent; White; Yellow
Shadows	No; Yes
Light location	Back; Front; Left; Right
Light color	Cyan; Green; White; Yellow
Body	Having a body; Having only shadows
Furniture	Having extra furniture; Having no furniture
Floor texture	Brick floor texture; Brighter floor; Darker floor
Wall texture	Gray brick texture; No wall texture; Red brick texture
Scale	Not having a scale; Having a scale

**Table 2:** The number of underestimates, accurate estimates, and overestimates at each investigated distance.

	25 cm	40 cm	55 cm	70 cm	85 cm	100 cm	115 cm	130 cm	145 cm	160 cm
DD, UE	129	132	114	123	115	87	94	76	95	71
DD, AE	25	54	114	79	91	137	113	127	118	148
DD, OE	160	128	86	112	108	90	107	111	101	95
GVR, UE	72	80	72	80	68	58	70	55	52	45
GVR, AE	20	34	56	45	52	71	51	62	66	66
GVR, OE	52	30	16	19	24	15	23	27	26	33

## Data Availability

The data presented in this study are available on request from the corresponding author.
